# Physiological Errors of Teetotalism

**Published:** 1856-03

**Authors:** 


*Physiological Errors of Teetotalism.*—Believing that the following remarks will be read with interest by your subscribers, both medical and general, and that they will tend to form correct views on a subject of the highest importance, I shall be glad if you will grant them a place in the *Lancet.*

Those who are in the habit of reading the able and logical articles of the *Westminster Review* must have been specially struck with one more than usually clever and logical, entitled, "*The Physiological Errors of Teetotalism*" in the July number. For the sake of those who do not read the *Review,* and who probably would never see the article, I have attempted to give a sort of digest of the writer's views and arguments, and, as far as possible, in his own words; so that I wish to take no credit for what I have done beyond what is due to me for the tedium of copying, and the choice and arrangement of the more strikng passages.

The two pillars on which teetotalism rests are—

1. That alcohol is a poison, and not a food.

2. That what is true of the excessive use of alcohol is true also, in proportionate degree, of the moderate and occasional use; in other words, that alcohol is *essentially* a poison.

On these, the whole argument for teetotalism and the whole of Dr. Carpenter's prize essay rest. The conclusions which the writer of the article establishes are—

1. That alcohol *is* food.

2. That use is *not* the same as abuse in the case of alcohol, of which it may be asserted that *quantitative* difference produces *qualitative* difference.

And thus the first pillar of teetotalism is shown to be a *scientific error,* and the second a *fallacy*; and, the pillars being destroyed, the entire system falls to the ground. From this, the nature and extent of the writer's task will be clearly understood; but I fear that the able manner in which he has performed it will not be so evident in my abridgment as it is in the original article.

Dr. Carpenter says, "the action of alcohol upon the animal body in health is *essentially poisonous;*" this proposition he attempts to prove, by showing that, "a soldier died on the spot, from drinking seven pints of brandy." Such examples prove that alcohol will kill, but not that it is *essentially* a poison. Life-giving oxygen might as easily be proved a poison. The fallacy which misleads Dr. Carpenter and his followers, is, the *assumption* that whatever is true of a large dose, is true, in a minor degree, of a small dose. There are cases in which what is true of the whole is true of a part; what is true of a large quantity is true of a small quantity; but there are many cases in which no such proportionate gradation exists—cases where *quantitative* difference produces *qualitative* difference; as, for instance, when a certain weight will make a steel spring *bend,* and a slight increase of the weight will make it *break;* in the one case, the force of cohesion is not destroyed; the spring, released from the weight, rebounds into its original shape; in the other, a slight addition altogether changes the effect, the spring breaks and cannot return to its original condition. People forget how frequently questions of *degree* involve questions of *kind.* Ice and steam differ only in the degree of heat; the cold of the arctics and the heat of the tropics are but differences of degree. Light, which is the necessary stimulus to the eye, produces blindness in excess. It will be objected that these are *natural,* and that alcohol is unnatural; but study is equally unnatural; and because men ruin their health by over-study we are not to conclude that all study, however moderate, is gradually fatal in its effects. Arsenic is no more natural than alcohol; yet when taken regularly, in minute doses, it gives both to men and horses increased vigour, increased beauty, and an enviable rejuvenescence. Theine, in doses of three or four grains, produces the most agreeable effects; double the quantity will be followed by unpleasant and dangerous symptoms. Granting, then, that in *some* cases whatever is true of excess, is *not* true of moderation; any case in which it *is* true, must depend upon intrinsic *qualities,* not affected by relations of *quantity.* Oxygen is not intrinsically a deleterious stimulus, but only quantitatively so. Lying is a vice, a vice qualitative; and there is not much difference, morally, between a man who lies liberally, and a man who is constantly but minutely mendacious. We are thus brought round to a consideration of the scientific error so intimately connected with the logical fallacy just exposed—the error, namely, of calling alcohol a poison. We hope to make it clear, that alcohol is not poison, but food. To the popular mind, it would be equally paradoxical to say iron is food, salt is food, chalk is food; the popular idea of food being limited to substances which, eaten by themselves, nourish and allay hunger.—*Mr. Hooper in Lancet.*

Concurring in these views generally, we have some hesitation in giving publicity to them, lest by so doing we should contribute to arguments tending toward an encouragement of intemperance. If, however, alcohol is "food," it must be admitted that it is a dangerous one, and totally unnecessary where simple nutritious materials are at hand, and where youth and rude health render any such auxiliary unnecessary.—*Dublin Medical Press.*

